# Accuracy Improvement Capability of Advanced Projectile Based on Course Correction Fuze Concept

**DOI:** 10.1155/2014/273450

**Published:** 2014-07-03

**Authors:** Ahmed Elsaadany, Yi Wen-jun

**Affiliations:** Nanjing University of Science and Technology, Nanjing 210094, China

## Abstract

Improvement in terminal accuracy is an important objective for future artillery projectiles. Generally it is often associated with range extension. Various concepts and modifications are proposed to correct the range and drift of artillery projectile like course correction fuze. The course correction fuze concepts could provide an attractive and cost-effective solution for munitions accuracy improvement. In this paper, the trajectory correction has been obtained using two kinds of course correction modules, one is devoted to range correction (drag ring brake) and the second is devoted to drift correction (canard based-correction fuze). The course correction modules have been characterized by aerodynamic computations and flight dynamic investigations in order to analyze the effects on deflection of the projectile aerodynamic parameters. The simulation results show that the impact accuracy of a conventional projectile using these course correction modules can be improved. The drag ring brake is found to be highly capable for range correction. The deploying of the drag brake in early stage of trajectory results in large range correction. The correction occasion time can be predefined depending on required correction of range. On the other hand, the canard based-correction fuze is found to have a higher effect on the projectile drift by modifying its roll rate. In addition, the canard extension induces a high-frequency incidence angle as canards reciprocate at the roll motion.

## 1. Introduction

Unguided projectiles show large missed distances on impact, even at relatively short ranges, due to wind and other meteorological conditions, muzzle velocity error, aiming error, and other factors affecting the projectile path during flight. For this reason, the improvement in terminal accuracy is a very important objective for future artillery projectiles. Generally it is often associated with range extension. Various concepts and modifications are proposed to correct the range and drift of artillery projectile like course correction fuze. The course correction fuze (CCF) concepts could provide an attractive and cost-effective solution for munitions accuracy improvement. The German TCF (trajectory correction fuze) concept uses an umbrella-shaped drag brake [1]. The United States LCCM (Low-Cost Competent Munitions) program uses four D-rings mounted on sliding rails [2, 3]. All these fuzes fit into the standard NATO two-inch thread of a conventional 155 mm projectile. Other CCF concepts consider canards based-correction to offer both range and drift correction. Canards based-correction is a common control mechanism used in smart projectiles that provide maneuver capability and range extension [4–7]. Typically, relatively small canards mounted on the front of the conventional projectile provide sufficient control authority to enable accurate flight trajectory. Deployed canards create changes in the overall aerodynamic characteristics of the projectile body, which most notably affect the aerodynamic roll and pitch damping. Costello [8, 9] studied and evaluated the potential of extending the range of a field artillery projectile using moveable canards. Generally, it is concluded that the improvement of artillery projectile accuracy is often associated with range extension. But range extension requires additional means, such as propellers and flight control systems which considerably affect the global cost.

In this paper, a six-degree-of-freedom (6-DOF) nonlinear model, in the atmospheric flight, is derived to predict the dynamic behavior of an advanced artillery projectile. The model is developed based on Newton's equations of motion, taking into consideration the influence of the Earth's rotation and ellipsoidal shape, Magnus effect, and the atmospheric wind. Furthermore, a modified standard atmospheric model to simulate air density and the speed of sound is used. The aerodynamic forces and moments of the projectile body and lifting canards are a function of both Mach number and angle of attack. This is followed with the definition of trajectory correction using two kinds of course correction modules: one is devoted to range correction (drag ring brake) and the second is devoted to drift correction (canard based-correction fuze). The concepts development and evaluation are performed using a 6-DOF model of a typical 155 mm spin-stabilized artillery projectile as a case study for the analysis. The analysis of the simulation results is discussed and shows that the impact accuracy of a conventional projectile using these course correction modules can be improved.

## 2. Projectile Dynamic Model

The motion of a projectile in atmospheric flight is assumed to be adequately described with six rigid body degrees of freedom comprised of three inertial position body coordinates as well as three Euler angle body attitudes. This section presents the equation of motion that models the projectile's atmospheric trajectory according to a set of initial launch conditions, as well as the methodology used to compute forces and moments acting on the projectile body [10–13]. The projectile is assumed to be both rigid (nonflexible) and rotationally symmetric about its spin axis. The translation dynamics give the projectile linear velocity as a result of the externally applied forces, whereas the rotation dynamics give its angular velocity as a function of the corresponding moments. The translational dynamic equations are obtained using a force balance equation on the projectile written in the body-fixed frame, given by

(1) $$\begin{array}{c} \left\{\begin{array}{c} \dot{u}\\ \dot{v}\\ \dot{w} \end{array}\right\}=\left\{\begin{array}{c} a_{x}^{b}\\ a_{y}^{b}\\ a_{z}^{b} \end{array}\right\}=\frac{1}{m}\left\{\begin{array}{c} F_{X}\\ F_{Y}\\ F_{Z} \end{array}\right\}-\left[\begin{array}{ccc} 0 & -r & q\\ r & 0 & -p\\ -q & p & 0 \end{array}\right]\left\{\begin{array}{c} u\\ v\\ w \end{array}\right\}. \end{array}$$

Note that the terms *F*_*X*_, *F*_*Y*_, and *F*_*Z*_ are the sum of weight, Magnus, and aerodynamic forces resolved in the body-fixed reference frame. The rotational dynamic equations are obtained using a moment equation about the projectile mass center and written in the body-fixed frame, given by

(2) $$\begin{array}{c} \left\{\begin{array}{c} \dot{p}\\ \dot{q}\\ \dot{r} \end{array}\right\}={\left[I\right]}^{-1}\left[\left\{\begin{array}{c} L\\ M\\ N \end{array}\right\}-\left[\begin{array}{ccc} 0 & -r & q\\ r & 0 & -p\\ -q & p & 0 \end{array}\right]\left[I\right]\left\{\begin{array}{c} p\\ q\\ r \end{array}\right\}\right]. \end{array}$$

Note that the terms *L*, *M*, and *N* are the sum of steady aerodynamic, unsteady aerodynamic, and Magnus moments resolved in the body-fixed reference frame. The rotational kinematic equations relate derivatives of Euler angles to angular velocity states which are given by

(3) $$\begin{array}{c} \left\{\begin{array}{c} \dot{\varphi }\\ \dot{\theta }\\ \dot{\psi } \end{array}\right\}=\left[\begin{array}{ccc} 1 & \sin\varphi \;\tan\theta & \cos\varphi \;\tan\theta \\ 0 & \cos\varphi & -\sin\varphi \\ 0 & \sin\varphi \;\text{sec}\;\theta & \cos\varphi \;\text{sec}\;\theta \end{array}\right]\left\{\begin{array}{c} p_{t}\\ q_{t}\\ r_{t} \end{array}\right\}, \end{array}$$

where ${\left[\begin{array}{ccc} p_{t} & q_{t} & r_{t} \end{array}\right]}^{T}$ are the angular acceleration components taking in account the error resulting from the Earth's rotation that was expressed in terms of the vehicle-carried north east down (NED) velocity and given by

(4) $$\begin{array}{c} \left\{\begin{array}{c} p_{t}\\ q_{t}\\ r_{t} \end{array}\right\}=\left\{\begin{array}{c} p\\ q\\ r \end{array}\right\}-J_{\text{BE}}\left[\begin{array}{c} \left(\omega^{e}+\dot{\mu }\right)\cos\lambda \\ -\dot{\lambda }\\ -\left(\omega^{e}+\dot{\mu }\right)\sin\lambda \end{array}\right],\\ J_{\text{BE}}=\left[\begin{array}{ccc} \cos\theta \cos\psi & \sin\varphi \sin\theta \cos\psi -\cos\varphi \sin\psi & \cos\varphi \sin\theta \cos\psi +\sin\varphi \sin\psi \\ \cos\theta \sin\psi & \sin\varphi \sin\theta \sin\psi +\cos\varphi \cos\psi & \cos\varphi \sin\theta \sin\psi -\sin\varphi \cos\psi \\ -\sin\theta & \sin\varphi \cos\theta & \cos\varphi \cos\theta \end{array}\right]. \end{array}$$

The derivative of the geodetic position can be expressed in terms of the vehicle-carried NED velocity and given by

(5) λ˙=VN(Rmer+h),μ˙=VE(Rnorm⁡+h)cos⁡λ,h˙=−VD.

The derivatives of the vehicle-carried NED velocity components taking in account Earth's rotation are, respectively, given by

(6) V˙N=−(μ˙+2ωe)sinλVE+λ˙VD+aN,V˙E=(μ˙+2ωe)sinλVN+(μ˙+2ωe)cos⁡λVD+aE,V˙D=−λ˙VN−(μ˙+2ωe)cos⁡λVE+aD.

The translational kinematic equations, relating vehicle-carried NED acceleration states to body-fixed acceleration states, are given by

(7) $$\begin{array}{c} \left\{\begin{array}{c} a_{N}\\ a_{E}\\ a_{D} \end{array}\right\}=J_{\text{BE}}^{-1}\;\left\{\begin{array}{c} a_{x}^{b}\\ a_{y}^{b}\\ a_{z}^{b} \end{array}\right\}, \end{array}$$

where *J*_BE_^−1^ is the inverse of the transformation matrix which rotates from the body frame to the vehicle-carried NED frame.

The parameters of equations from (5) to (6) are defined and derived based on the WGS 84 (world geodetic system 84, which was originally proposed in 1984 and lastly updated in 2004) [14].

### 2.1. Force and Moment Model for Projectile Body

During flight there are two kinds of forces acting on projectile motion. They are weight and resultant body and canards aerodynamic forces. When the aerodynamic forces do not pass through the center of gravity, moments are created. The aerodynamic forces and moments are calculated within the flight simulation using the aerodynamic coefficients that were predicted using PRODAS program. The total forces acting on the projectile can be expressed as

(8) {FXFYFZ}={FWXFWYFWZ}+{FAXFAYFAZ}+{FMXFMYFMZ}+{FCXFCYFCZ},

(9) {FWXFWYFWZ}=mg[−sinθsinφcos⁡θcos⁡φcos⁡θ],

(10) {FAXFAYFAZ}=−q−S(CD[100]+CNα[0sin⁡(β)tan⁡(α)]),

(11) {FMXFMYFMZ}=q−S(ptDVt)CYpα[0tan⁡(α)−sin⁡(β)],

where *V*_*t*_ is the body velocity magnitude and is given by

(12) $$\begin{array}{c} V_{t}=\sqrt{u^{2}+v^{2}+w^{2}.} \end{array}$$

The lateral and longitudinal aerodynamic angles of attack, respectively, are computed as

(13) $$\begin{array}{c} \sin\left(\beta \right)=\left(\frac{v}{V_{t}}\right),\;\;\tan\left(\alpha \right)=\left(\frac{w}{u}\right). \end{array}$$

These forces are expressed in the body-fixed frame and split into contributions due to weight (*W*), body, and canard (*C*) aerodynamic forces, respectively. The body aerodynamic forces split into a standard aerodynamic (*A*) and Magnus (*M*) forces, respectively. The total moments acting on the projectile can be expressed by

(14) {LMN}={LSAMSANSA}+{LUAMUANUA}+{LMMMNM}+{LCMCNC},{LSAMSANSA}=[0−RCPzRCPyRCPz0−RCPx−RCPyRCPx0]{FAXFAYFAZ},{LUAMUANUA}=q−SD(Cmα[0tan(α)−sin(β)]+(DVt)Cmq[0qtrt]     +(ptDVt)Clp[100]),{LMMMNM}=q−SD(ptDVt)Cnpα[0sin⁡(β)tan⁡(α)].

These moments contain steady aerodynamic (SA), unsteady aerodynamic (UA), Magnus (*M*), and canard aerodynamic moments (*C*), respectively. The steady aerodynamic moment is computed with a cross product between the distance vector from the center of gravity to the body center of pressure and the body aerodynamic force vector in (10). The aerodynamic coefficients and the distance of aerodynamic center are all a function of local Mach number at the mass center of the projectile. Computationally, these Mach number dependent parameters are obtained by a table look-up scheme using linear interpolation. Expressions for the canard forces and moments are derived in the following.

### 2.2. Force and Moment Model for Canard

**Figure 1:**
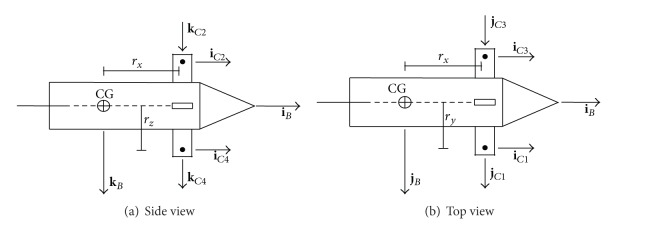
Basic geometrical data of the proposed projectile.

The canard aerodynamic force and moment are modeled based on [8]. The total canard aerodynamic force and moment are the sum of individual force and moment produced by each lifting canard and given by

(15) $$\begin{array}{c} F_{C}=\overset{N_{c}}{\underset{i=1}{\sum }}F_{CXi}i_{B}+\overset{N_{c}}{\underset{i=1}{\sum }}F_{CYi}j_{B}+\overset{N_{c}}{\underset{i=1}{\sum }}F_{CZi}k_{B},\\ \tau_{C}=\overset{N_{c}}{\underset{i=1}{\sum }}L_{Ci}i_{B}+\overset{N_{c}}{\underset{i=1}{\sum }}M_{Ci}j_{B}+\overset{N_{c}}{\underset{i=1}{\sum }}N_{Ci}k_{B}, \end{array}$$

where *N*_*c*_ is the number of lifting canards. This work uses four lifting canards.  Figure 1 shows a diagram of the canards used in this development. The relative aerodynamic velocity components of the *i*th canard are calculated according to

(16) $$\begin{array}{c} u_{ci}=u-{rr}_{yi}+qr_{zi},\\ v_{ci}=v+{rr}_{xi}-pr_{zi},\\ w_{ci}=w-{qr}_{xi}+pr_{yi}, \end{array}$$

where *r*_*xi*_, *r*_*yi*_, and *r*_*zi*_ are the vector components from the projectile center of gravity to the computation point on the *i*th lifting canard resolved in the body frame. The lift and drag forces produced by the *i*th canard are given by

(17) $$\begin{array}{c} L_{Ci}=\frac{1}{2}\rho \left(u_{ci}^{2}+v_{ci}^{2}+w_{ci}^{2}\right)S_{Ci}\;C_{Lci},\\ D_{Ci}=\frac{1}{2}\rho \left(u_{ci}^{2}+v_{ci}^{2}+w_{ci}^{2}\right)S_{Ci}\;C_{Dci},\\ C_{Lci}=C_{lc}{\left(M_{Ci}\right)\alpha }_{Ci},\\ C_{Dci}=C_{Dc}\left(M_{Ci}\right), \end{array}$$

where *S*_*ci*_ is the *i*th canard reference area. *C*_*lc*_ and *C*_*Dc*_ are the canard lift and drag aerodynamic coefficients, respectively. *α*_*Ci*_ is the aerodynamic angle of attack of the *i*th canard. The canard lift and drag aerodynamic coefficients are Mach number dependent. Computationally, they are obtained by a table look-up scheme using linear interpolation. The Mach number is calculated at the computation point of each canard as follows:

(18) $$\begin{array}{c} M_{Ci}=\frac{\sqrt{u_{ci}^{2}+v_{ci}^{2}+w_{ci}^{2}}}{a}. \end{array}$$

**Figure 2:**
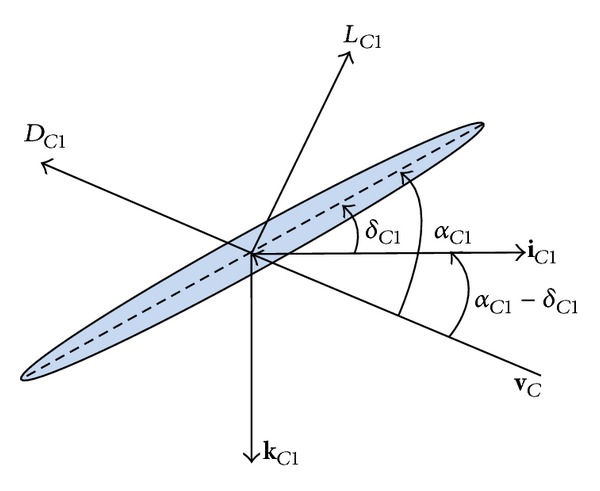
Canard aerodynamic model force diagram.

In the following section, only the total resultant force and moment equations for canard 1 are expressed, whereas all other canards can be computed in the same manner with suitable modifications. The canard angle of attack is computed in the same manner as the body angle of attack except for the local relative velocity at the canard computation point which is used as follows:

(19) $$\begin{array}{c} \alpha_{C1}=\delta_{c1}+\tan^{-1}\left(\frac{w_{c1}}{u_{c1}}\right), \end{array}$$

where *δ*_*c*1_ is the deflection angle of canard 1. The aerodynamic force and moment due to canard 1, as shown in Figure 2, are given by

(20) {FCX1FCY1FCZ1}={LC1wc1uc12+wc12−DC1uc1uc12+wc120−LC1uc1uc12+wc12−DC1wc1uc12+wc12},{LC1MC1NC1}={FCZ1ry1−FCY1rz1FCX1rz1−FCZ1rx1FCY1rx1−FCX1ry1}.

## 3. Atmospheric Model

**Figure 3:**
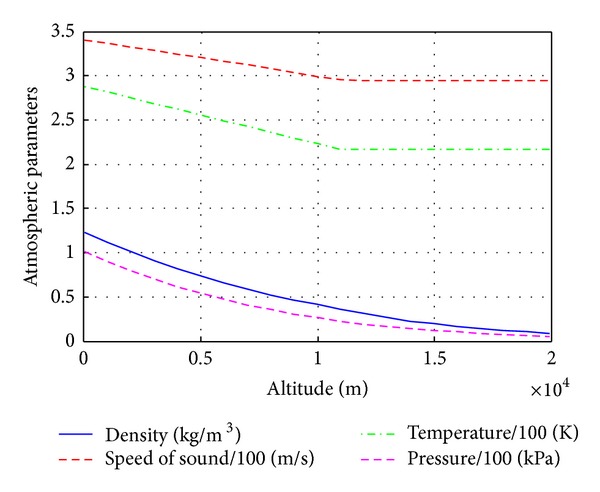
Atmospheric parameters versus altitude.

Variations in meteorological conditions have an effect on the projectile traveling through the atmosphere and hence affect its trajectory. The artillery projectile typically has peak altitudes of about 20 kilometers which is within the troposphere and is thus subjected to air density and drag. With increasing altitude, air properties such as density, temperature, pressure, and air viscosity change. Therefore, this changing is taken into consideration during the trajectory calculation to get an accurate prediction. For this purpose, a standard atmosphere model is developed based on the International Standard Atmosphere (ISA) [15]. Expressions for air density, *ρ*, and speed of sound, *a*, as a function of altitude, *Z*, can be derived and are given by

(21) $$\begin{array}{c} \rho =\rho_{0}{\left(\frac{T}{T_{0}}\right)}^{\left((g/RL)-1\right)}\exp\left(\frac{g\left(Z_{\text{trop}}-Z\right)}{RT}\right),\\ a=\sqrt{\gamma RT,} \end{array}$$

where *T* is the air temperature and is given by

(22) $$\begin{array}{c} T=T_{0}-LZ, \end{array}$$

where *ρ*_0_ and *T*_0_ are the air density and air temperature at the sea level, respectively. *g*, *R*, *L*, *Z*_trop_, and *γ* are gravity acceleration, real gas constant for air, temperature lapse rate, tropopause altitude, and adiabatic gas constant, respectively.  Figure 3 depicts the variation of the air density, sound speed, air temperature, and air pressure as a function of altitude.

## 4. Wind Model

**Figure 4:**
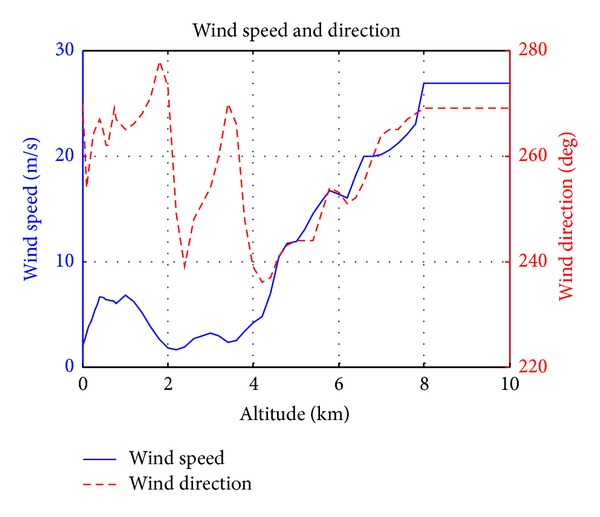
Wind speed and direction versus altitude.

The Earth's atmosphere generally fosters air velocity perturbations that can significantly modify the trajectory of a projectile. It is common to separate the atmospheric air velocity perturbations into steady and turbulence components, typically called mean wind and atmospheric turbulence, respectively. The atmospheric wind model in the current work is adopted based on a real recorded data that had been used in this work [16].  Figure 4 shows a recorded data for wind speed (in m/s) and direction (in degrees) as a function of altitude.

## 5. Physical Properties

The developed nonlinear 6-DOF model in this work uses the nominal physical properties of a typical 155 mm spin-stabilized projectile. These physical properties are listed in Table 1.

## 6. Aerodynamic Coefficients

**Figure 5:**
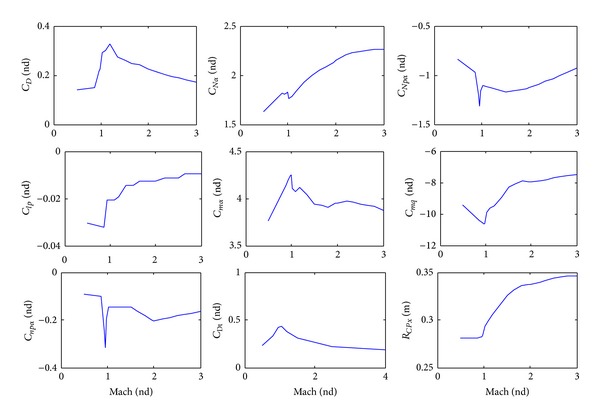
Body aerodynamic parameters versus Mach number.

**Figure 6:**
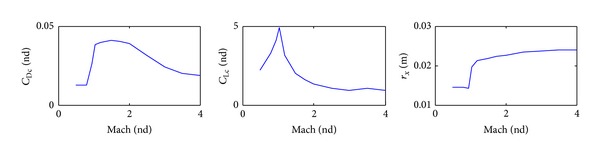
Canard aerodynamic parameters versus Mach number.

The body nondimensional aerodynamic coefficients and distance that were predicted using PRODAS program, based on the dimensions of the nominal 155 mm projectile (Table 1), are shown in Figure 5. The total drag coefficient (body + drag ring brake), in case of range correction, is also shown in Figure 5. The aerodynamic coefficients and distance, for one pair of the proposed canards, are shown in Figure 6. In the case of using course correction concepts, the presence of their aerodynamic coefficients is considered only in the external force and moment terms in the 6-DOF equations of motion.

## 7. Simulation Conditions

Trajectory simulations have been performed in the following flight conditions:

muzzle velocity, *V*_0_ = 910 [m/sec], and initial elevation angle, *θ*_0_ = 45°.

### 7.1. Muzzle Spin Rate Estimation

According to McCoy definition [13], the muzzle spin rate can be estimated by

(23) $$\begin{array}{c} p_{0}=\frac{2\pi V_{0}}{\eta D}\;\left[\text{rad}/\text{sec}\right], \end{array}$$

where *η* is the rifling twist rate at the gun muzzle (caliber per turn).

## 8. Trajectory Correction

**Figure 7:**
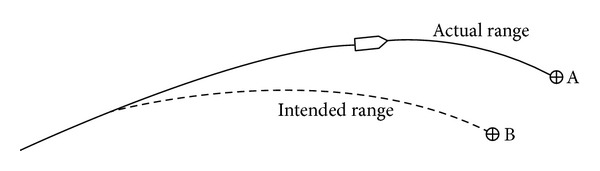
The intended and actual paths of the projectile.

**Figure 8:**
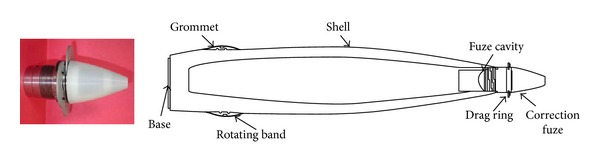
Drag ring brake module.

**Figure 9:**
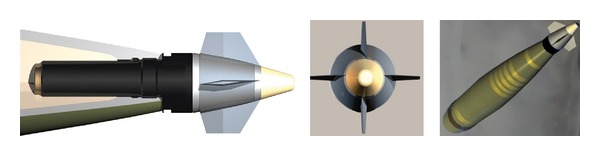
Canard based-correction fuze.

The unguided projectiles show large missed distances on impact, even at relatively short ranges. As shown in Figure 7, the projectile is fired at intended target B, but, due to wind and other meteorological conditions, muzzle velocity error, aiming error, and so forth, the projectile actually impacts point A. For this reason, several concepts and modifications, for trajectory correction, have been proposed. In this work, the trajectory correction is obtained by using two kinds of modules, one is devoted to range correction (drag ring brakes, Figure 8) and the second is devoted to drift correction (canard based-correction fuze, Figure 9).

### 8.1. Range Correction

The concept of drag ring brake has been discussed by Hollis and Brandon [2], which replaces the standard fuze of conventional artillery projectile with trajectory corrector module, without changes within the ogive shape of the artillery projectile. Drag brakes are designed to fit onto a spin-stabilized projectile within a fuze which screws into the forward part of the projectile (Figure 8).

**Figure 10:**
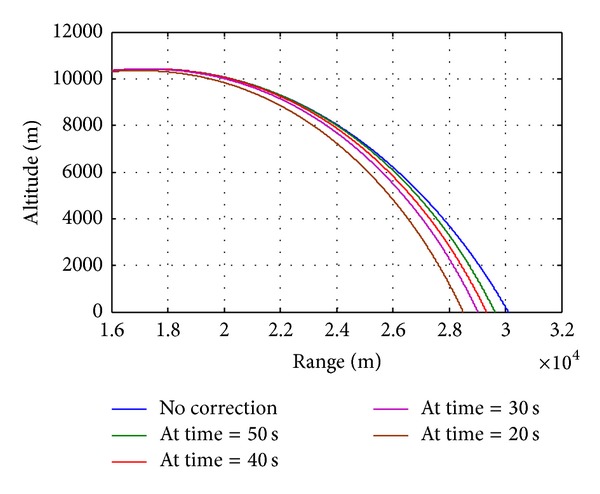
Altitude versus range.

**Figure 11:**
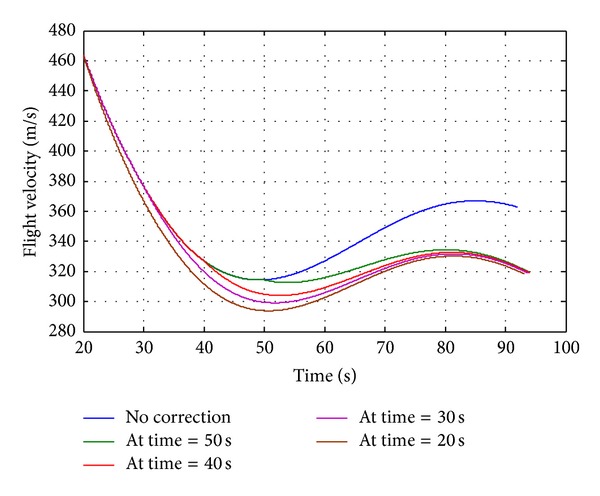
Flight velocity versus time.

During the course correction phase, semicircular plates will deploy from the module. The plates create a blunt cross-sectional area in front of the projectile, thus creating more drag and effectively slowing the projectile. The analysis is carried out for deploying the drag brake at various stages of trajectory and once the drag ring module is deployed, it will remain open throughout the trajectory, and then the projectile will slow down, ultimately bringing it closer to the intended target. The results show that the drag ring brakes are found to be highly capable for range correction. The deploying of the drag brake in early stage of trajectory results in maximum range correction (Figure 10). Hence, maximum range correction is observed at the earliest occasion time, where occasion time of 20 seconds gives correction of 1609 m (i.e., 5.34% of the total nominal range). The differences in velocities due to drag brakes are shown in Figure 11. It is observed that deployment of drag brake throughout the flight reduces the remaining velocity, by nearly 44 m/sec, which gives the required correction in range. From the results listed in Table 2, we can conclude that the occasion time can be predefined depending on required correction of range.

### 8.2. Drift Correction

**Figure 12:**
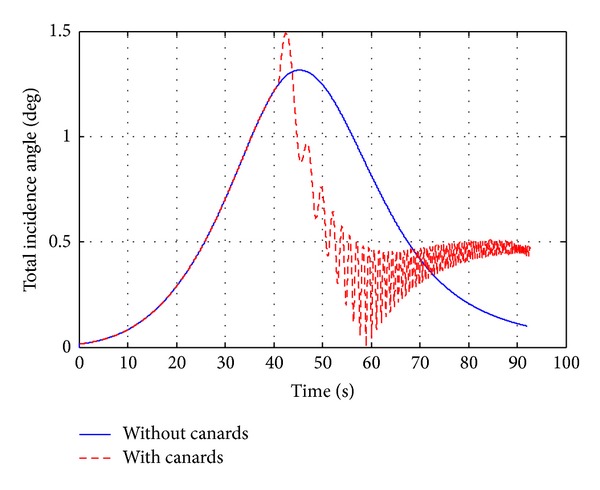
Total incidence angle, *α*_*t*_, versus time.

**Figure 13:**
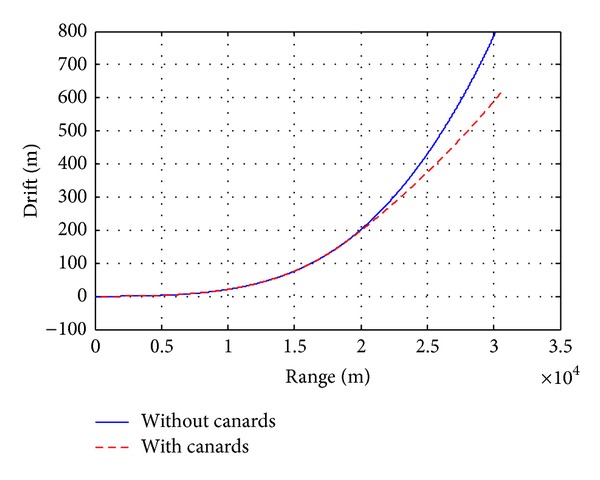
Drift versus range.

**Figure 14:**
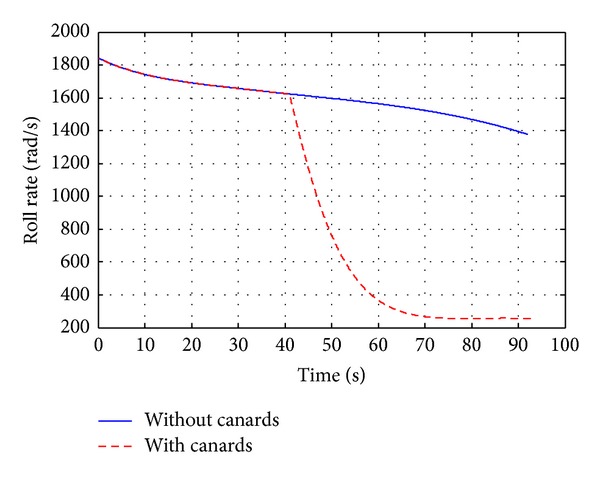
Body roll rate versus time.

**Figure 15:**
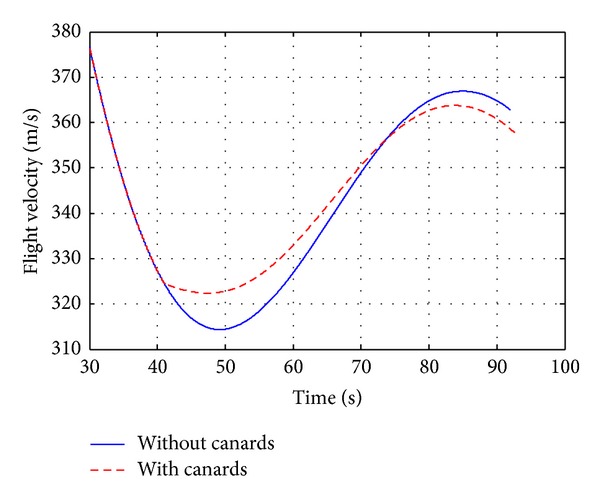
Flight velocity versus time.

A canard based-correction fuze (see Figure 9) has been characterized by aerodynamic computations and 6-DOF flight dynamics investigations in order to analyze the effects on deflection of the projectile aerodynamic parameters. The drift correction is accomplished in this work with a four equally spaced canards and each of which has a reference area of 18.63  cm^2^. Figures 12–15 show computational results contrasting the nominal and corrected trajectories for the proposed projectile in the flight conditions described above. Note that the four canards are deployed at the apex of flight.

Figure 12 shows the total incidence angle of the projectile in both the nominal and canards based cases. The maximum nominal incidence angle is nearly 1.3°, while the canards induce maximum incidence angle oscillations of 1.5°. However, note the high-frequency incidence angle oscillations that occur in the canards based case as canards reciprocate at the roll frequency. Figure 12 shows the behavior of the projectile total incidence angle *α*_*t*_ that is calculated by

(24) $$\begin{array}{c} \sin\alpha_{t}=\sqrt{{\left(\cos\beta \sin\alpha \right)}^{2}+{\left(\sin\beta \right)}^{2}.} \end{array}$$

Figure 13 demonstrates that the canard based trajectory is successful in reducing drift error, recording drift of less than 615 m, compared with the nominal case which recorded drift of 800 m.  Figure 14, showing the projectile roll rate profiles, demonstrates the slight increase in roll damping that occurs during canards extension, where the roll rate is reduced from 1845 rad/sec at the apex of flight to 257 rad/sec within nearly 30 sec. In Figure 15, the total velocity-time histories varied only on the order of a 2.7%, demonstrating that the drag penalty associated with canards of this size is relatively small.

## 9. Conclusion

Future artillery systems will require guided projectiles for accuracy. This paper summarized an analysis on CCF options to improve the terminal accuracy of conventional spin-stabilization projectile. Two kinds of course correction modules are used, one is devoted to range correction (drag ring brakes) and the second is devoted to drift correction (four canards based-correction fuze). The correction modules performance is analyzed and evaluated through a 6-DOF nonlinear model of a typical 155 mm spin-stabilized artillery projectile. The simulation results show that the impact accuracy, using these course correction modules, can be improved, whereas the drag ring brakes are found to be highly capable for range correction, and the deploying of it in early stage of trajectory results in maximum range correction, where deployment time of 20 seconds gives correction of 1609 m (i.e., about 5.34% of the total nominal range). However, the deployment time can be predefined depending on the required correction of range. Also, it is observed that deployment of drag brake throughout the flight reduces the remaining flight velocity which gives the required correction in range. On the other hand, the canard based-correction concept can rapidly reduce the roll rate from 1845 rad/sec to 257 rad/sec within 30 seconds that dramatically decreases the drift by 185 m (i.e., about 23.13% from the nominal drift). In addition, the canard extension induces a high-frequency incidence angle as canards reciprocate at the roll motion. Note that these oscillations occur in the canards based case as canards reciprocate at the roll frequency. Also, it is observed that the total velocity-time histories varied only on the order of a 2.7%, demonstrating that the drag penalty associated with canards of this size is relatively small.

## Figures and Tables

**Table 1:** Projectile physical properties.

Parameter	Value
Caliber	*D* = 155 mm
Length	*l* = 902 mm
Total mass	*m* = 45 kg
Center of gravity from the nose	*X*_CG_ = 558 mm
Axial moment of inertia	*I*_*XX*_ = 0.162 Kg*·*m^2^
Lateral moment of inertia	*I*_*YY*_ = *I*_*ZZ*_ = 1.763 Kg*·*m^2^

**Table 2:** Correction versus occasion time.

Ctrl start time [sec]	Correction [m]
*t* = 20	1609
*t* = 30	1084
*t* = 40	780
*t* = 50	480

## Nomenclature

### A List of Symbols

*C*_*D*_: Drag force coefficient
*C*_*Nα*_: Normal force coefficient derivative
*C*_*Yp**α*_: Magnus force coefficient derivative
*C*_*lp*_: Damp in roll coefficient derivative
*C*_*mq*_: Pitching damping moment coefficient derivative
*C*_*mα*_: Overturning moment coefficient derivative
*C*_*np**α*_: Magnus moment coefficient derivative
*C*_*Dt*_: Total drag force coefficient (body and opened drag ring)
*C*_*DC*_: Canard drag force coefficient
*C*_*lC*_: Canard lift force coefficient
*D*: Projectile diameter (m)
*g*: Normal gravity on the ellipsoidal surface (m/sec^2^)
[*I*]: Mass moment-of-inertia matrix
*J*_BE_: Transformation matrix which rotates from geodetic frame into body frame
*m*: Total mass of projectile (kg)
*R*_mer_, *R*_norm⁡_: Meridian and normal radiuses of the Earth's curvature, respectively (m)
${\left[\begin{array}{ccc} p & q & r \end{array}\right]}^{T}\text{:}$: Body rotational rate vector (rad/s)
${\left[\begin{array}{ccc} a_{N} & a_{E} & a_{D} \end{array}\right]}^{T}\text{:}$: Acceleration vector acting on the body in geodetic frame (m/sec^2^)
${\left[\begin{array}{ccc} a_{x}^{b} & a_{y}^{b} & a_{z}^{b} \end{array}\right]}^{T}\text{:}$: Acceleration vector acting on the body in body frame (m/sec^2^)
${\left[\begin{array}{ccc} F_{X} & F_{Y} & F_{Z} \end{array}\right]}^{T}\text{:}$: Components of the total resultant force acting on the body (N)
${\left[\begin{array}{ccc} u & v & w \end{array}\right]}^{T}\text{:}$: Components of the velocity vector of the body in the body frame (m/s)
${\left[\begin{array}{ccc} V_{N} & V_{E} & V_{D} \end{array}\right]}^{T}\text{:}$: Components of the velocity vector of the body in the geodetic frame (m/s)
${\left[\begin{array}{ccc} L & M & N \end{array}\right]}^{T}\text{:}$: Components of the total resultant moment acting on the body (N*·*m)
$\overset{-}{q}\text{:}$: Dynamic pressure at the projectile mass center
*S*: Projectile reference area (m^2^)
*h*: Altitude of body C.G in the geodetic frame (m).

### Greek Symbols

*φ*, *θ*, *ψ*: Projectile roll, pitch and yaw angles, respectively (deg)
*β*, *α*: Lateral and longitudinal aerodynamic angles, respectively (rad)
*λ*, *μ*: Latitude and longitude of body C.G in the geodetic frame, respectively (rad)
*ω*^*e*^: Earth's rotation rate (rad/s).

### Subscripts

*o*: Initial values at the firing site.
